# Supplementary material for: Non-linear Functional Brain Co-activations in Short-Term Memory Distortion Tasks
Source: Front Neurosci. 2021 Dec 3;15:778242. doi: 10.3389/fnins.2021.778242 (PMC8678091; doi:10.3389/fnins.2021.778242)
Supplement: Supplementary file 1 [file Data_Sheet_1.DOCX]

Table S1. AAL atlas regions with corresponding MNI coordinates.

| Id | AAL atlas region | MNI coordinates | | |
| --- | --- | --- | --- | --- |
| 1 | Precentral_L | -38.65 | -5.68 | 50.94 |
| 2 | Precentral_R | 41.37 | -8.21 | 52.09 |
| 3 | Frontal_Sup_L | -18.45 | 34.81 | 42.2 |
| 4 | Frontal_Sup_R | 21.9 | 31.12 | 43.82 |
| 5 | Frontal_Sup_Orb_L | -16.56 | 47.32 | -13.31 |
| 6 | Frontal_Sup_Orb_R | 18.49 | 48.1 | -14.02 |
| 7 | Frontal_Mid_L | -33.43 | 32.73 | 35.46 |
| 8 | Frontal_Mid_R | 37.59 | 33.06 | 34.04 |
| 9 | Frontal_Mid_Orb_L | -30.65 | 50.43 | -9.62 |
| 10 | Frontal_Mid_Orb_R | 33.18 | 52.59 | -10.73 |
| 11 | Frontal_Inf_Oper_L | -48.43 | 12.73 | 19.02 |
| 12 | Frontal_Inf_Oper_R | 50.2 | 14.98 | 21.41 |
| 13 | Frontal_Inf_Tri_L | -45.58 | 29.91 | 13.99 |
| 14 | Frontal_Inf_Tri_R | 50.33 | 30.16 | 14.17 |
| 15 | Frontal_Inf_Orb_L | -35.98 | 30.71 | -12.11 |
| 16 | Frontal_Inf_Orb_R | 41.22 | 32.23 | -11.91 |
| 17 | Rolandic_Oper_L | -47.16 | -8.48 | 13.95 |
| 18 | Rolandic_Oper_R | 52.65 | -6.25 | 14.63 |
| 19 | Supp_Motor_Area_L | -5.32 | 4.85 | 61.38 |
| 20 | Supp_Motor_Area_R | 8.62 | 0.17 | 61.85 |
| 21 | Olfactory_L | -8.06 | 15.05 | -11.46 |
| 22 | Olfactory_R | 10.43 | 15.91 | -11.26 |
| 23 | Frontal_Sup_Medial_L | -4.8 | 49.17 | 30.89 |
| 24 | Frontal_Sup_Medial_R | 9.1 | 50.84 | 30.22 |
| 25 | Frontal_Med_Orb_L | -5.17 | 54.06 | -7.4 |
| 26 | Frontal_Med_Orb_R | 8.16 | 51.67 | -7.13 |
| 27 | Rectus_L | -5.08 | 37.07 | -18.14 |
| 28 | Rectus_R | 8.35 | 35.64 | -18.04 |
| 29 | Insula_L | -35.13 | 6.65 | 3.44 |
| 30 | Insula_R | 39.02 | 6.25 | 2.08 |
| 31 | Cingulum_Ant_L | -4.04 | 35.4 | 13.95 |
| 32 | Cingulum_Ant_R | 8.46 | 37.01 | 15.84 |
| 33 | Cingulum_Mid_L | -5.48 | -14.92 | 41.57 |
| 34 | Cingulum_Mid_R | 8.02 | -8.83 | 39.79 |
| 35 | Cingulum_Post_L | -4.85 | -42.92 | 24.67 |
| 36 | Cingulum_Post_R | 7.44 | -41.81 | 21.87 |
| 37 | Hippocampus_L | -25.03 | -20.74 | -10.13 |
| 38 | Hippocampus_R | 29.23 | -19.78 | -10.33 |
| 39 | ParaHippocampal_L | -21.17 | -15.95 | -20.7 |
| 40 | ParaHippocampal_R | 25.38 | -15.15 | -20.47 |
| 41 | Amygdala_L | -23.27 | -0.67 | -17.14 |
| 42 | Amygdala_R | 27.32 | 0.64 | -17.5 |
| 43 | Calcarine_L | -7.14 | -78.67 | 6.44 |
| 44 | Calcarine_R | 15.99 | -73.15 | 9.4 |
| 45 | Cuneus_L | -5.93 | -80.13 | 27.22 |
| 46 | Cuneus_R | 13.51 | -79.36 | 28.23 |
| 47 | Lingual_L | -14.62 | -67.56 | -4.63 |
| 48 | Lingual_R | 16.29 | -66.93 | -3.87 |
| 49 | Occipital_Sup_L | -16.54 | -84.26 | 28.17 |
| 50 | Occipital_Sup_R | 24.29 | -80.85 | 30.59 |
| 51 | Occipital_Mid_L | -32.39 | -80.73 | 16.11 |
| 52 | Occipital_Mid_R | 37.39 | -79.7 | 19.42 |
| 53 | Occipital_Inf_L | -36.36 | -78.29 | -7.84 |
| 54 | Occipital_Inf_R | 38.16 | -81.99 | -7.61 |
| 55 | Fusiform_L | -31.16 | -40.3 | -20.23 |
| 56 | Fusiform_R | 33.97 | -39.1 | -20.18 |
| 57 | Postcentral_L | -42.46 | -22.63 | 48.92 |
| 58 | Postcentral_R | 41.43 | -25.49 | 52.55 |
| 59 | Parietal_Sup_L | -23.45 | -59.56 | 58.96 |
| 60 | Parietal_Sup_R | 26.11 | -59.18 | 62.06 |
| 61 | Parietal_Inf_L | -42.8 | -45.82 | 46.74 |
| 62 | Parietal_Inf_R | 46.46 | -46.29 | 49.54 |
| 63 | SupraMarginal_L | -55.79 | -33.64 | 30.45 |
| 64 | SupraMarginal_R | 57.61 | -31.5 | 34.48 |
| 65 | Angular_L | -44.14 | -60.82 | 35.59 |
| 66 | Angular_R | 45.51 | -59.98 | 38.63 |
| 67 | Precuneus_L | -7.24 | -56.07 | 48.01 |
| 68 | Precuneus_R | 9.98 | -56.05 | 43.77 |
| 69 | Paracentral_Lobule_L | -7.63 | -25.36 | 70.07 |
| 70 | Paracentral_Lobule_R | 7.48 | -31.59 | 68.09 |
| 71 | Caudate_L | -11.46 | 11 | 9.24 |
| 72 | Caudate_R | 14.84 | 12.07 | 9.42 |
| 73 | Putamen_L | -23.91 | 3.86 | 2.4 |
| 74 | Putamen_R | 27.78 | 4.91 | 2.46 |
| 75 | Pallidum_L | -17.75 | -0.03 | 0.21 |
| 76 | Pallidum_R | 21.2 | 0.18 | 0.23 |
| 77 | Thalamus_L | -10.85 | -17.56 | 7.98 |
| 78 | Thalamus_R | 13 | -17.55 | 8.09 |
| 79 | Heschl_L | -41.99 | -18.88 | 9.98 |
| 80 | Heschl_R | 45.86 | -17.15 | 10.41 |
| 81 | Temporal_Sup_L | -53.16 | -20.68 | 7.13 |
| 82 | Temporal_Sup_R | 58.15 | -21.78 | 6.8 |
| 83 | Temporal_Pole_Sup_L | -39.88 | 15.14 | -20.18 |
| 84 | Temporal_Pole_Sup_R | 48.25 | 14.75 | -16.86 |
| 85 | Temporal_Mid_L | -55.52 | -33.8 | -2.2 |
| 86 | Temporal_Mid_R | 57.47 | -37.23 | -1.47 |
| 87 | Temporal_Pole_Mid_L | -36.32 | 14.59 | -34.08 |
| 88 | Temporal_Pole_Mid_R | 44.22 | 14.55 | -32.23 |
| 89 | Temporal_Inf_L | -49.77 | -28.05 | -23.17 |
| 90 | Temporal_Inf_R | 53.69 | -31.07 | -22.32 |


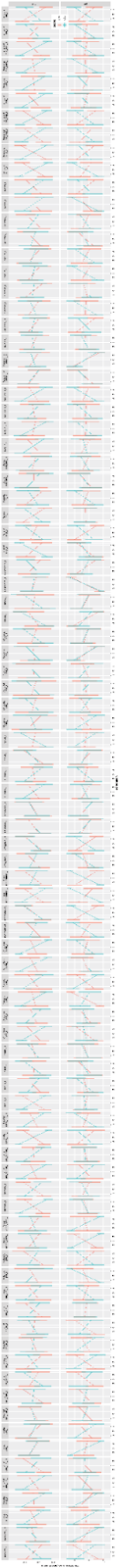


Figure S1. Results of the correlation × phase × probe × ROI interaction in the global processing task, averaged over condition levels. All ROIs are presented. The ROIs for which the contrast lure - positive yielded p < 0.05 (multiple-comparison corrected) are given in Table 2.


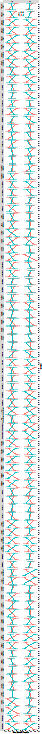


Figure S2. Results of the correlation × phase × probe × ROI interaction in the local processing task averaged over condition levels. All ROIs are presented. The ROIs for which the contrast lure - positive yielded p < 0.05, multiple-comparison corrected, are given in Table 3.


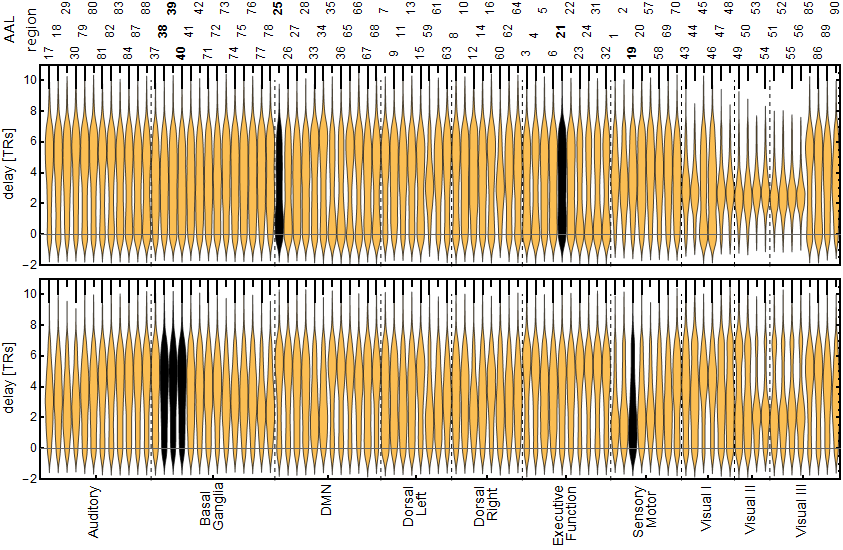


Figure S3. Distributions of delays between the stimuli and the nearest BOLD peaks for the global information processing task in (top panel) encoding and (bottom panel) retrieval phase. The 90 AAL brain areas (the numerical labels in the top correspond with the ID numbers in Table S1) are grouped according to which resting-state networks they belong to. The distributions plotted in black correspond to those reported as statistically significant in Table 2. Note the peculiar modes of the distributions in Visual areas as well as the bimodal distributions, for instance ROIs 31 and 32.


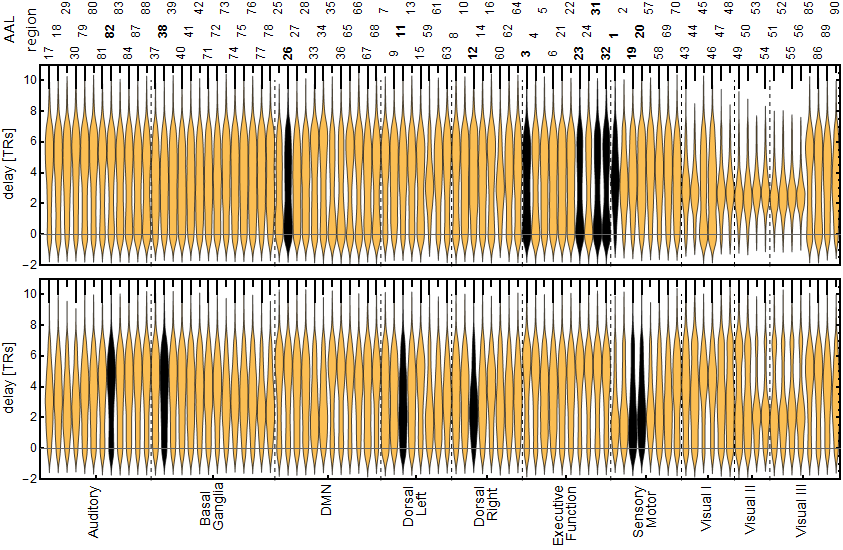


Figure S4. Distributions of delays between the stimuli and the nearest BOLD peaks for the *local* information processing task in (top panel) encoding and (bottom panel) retrieval phase. The 90 AAL brain areas (cf. Table S1) are grouped into resting-state networks. The distributions in black are the ones in bold in Table 3.
